# Hapln2 in Neurological Diseases and Its Potential as Therapeutic Target

**DOI:** 10.3389/fnagi.2019.00060

**Published:** 2019-03-21

**Authors:** Qinqin Wang, Chunmei Wang, Bingyuan Ji, Jiawei Zhou, Chunqing Yang, Jing Chen

**Affiliations:** ^1^Neurobiology Key Laboratory, Jining Medical University, Jining, China; ^2^State Key Laboratory of Neuroscience, Institute of Neuroscience, Center for Excellence in Brain Science and Intelligence Technology, Chinese Academy of Sciences, Shanghai, China; ^3^Division of Biomedical Sciences, Warwick Medical School, University of Warwick, Coventry, United Kingdom

**Keywords:** Hapln2, aggregates, Parkinson’s disease, Alzheimer’s disease, schizophrenia

## Abstract

Hyaluronan and proteoglycan link protein 2 (Hapln2) is important for the binding of chondroitin sulfate proteoglycans to hyaluronan. Hapln2 deficiency leads to the abnormal expression of extracellular matrix (ECM) proteins and dysfunctional neuronal conductivity, demonstrating the vital role of Hapln2 in these processes. Studies have revealed that Hapln2 promotes the aggregation of α-synuclein, thereby contributing to neurodegeneration in Parkinson’s disease (PD), and it was recently suggested to be in intracellular neurofibrillary tangles (NFTs). Additionally, the expression levels of Hapln2 showed lower in the anterior temporal lobes of individuals with schizophrenia than those of healthy subjects. Together, these studies implicate the involvement of Hapln2 in the pathological processes of neurological diseases. A better understanding of the function of Hapln2 in the central nervous system (CNS) will provide new insights into the molecular mechanisms of these diseases and help to establish promising therapeutic strategies. Herein, we review the recent progress in defining the role of Hapln2 in brain physiology and pathology.

## Introduction

Parkinson’s disease (PD), Alzheimer’s disease (AD) and schizophrenia are neurological diseases characterized by the dysfunction of certain types of neurons (Hardy and Higgins, 1992; Korth, 2012; Ghosh et al., 2016). However, the molecular mechanisms underlying the pathological processes of these brain disorders remain elusive. Accumulating evidence suggests that the pathogenesis of these diseases involve abnormal protein aggregates (Hardy and Higgins, 1992; Korth, 2012; Ghosh et al., 2016). For example, in PD, the formation of Lewy bodies comprising α-synuclein aggregates leads to the degeneration of dopaminergic neurons in the substantia nigra (SN; Ghosh et al., 2016; Wang et al., 2016). The deposition of amyloid beta protein induces neuronal cell death in the development of AD (Hardy and Higgins, 1992). Recently, schizophrenia has been linked with the abnormal deposition of disrupted in schizophrenia 1 (DISC1) aggregates (Atkin and Kittler, 2012; Korth, 2012). Moreover, all of these three diseases have been linked with the dysfunction of the ubiquitin-proteasome pathway (UPP; Lam et al., 2000; Bousman et al., 2010; Shen et al., 2013), which balances protein synthesis with degradation (Tsukamoto and Yokosawa, 2006). Substrates for degradation within this pathway are specified by E3 ubiquitin ligases (Ardley and Robinson, 2005), and we recently demonstrated that the E3 ubiquitin ligases Hrd1, Gp78 and Parkin colocalized with hyaluronan and proteoglycan link protein 2 (Hapln2; Wang et al., 2016).

Studies have shown that Hapln2, also known as brain-derived link protein1 (Bral1), is vital for neuronal conductivity and the formation of the extracellular matrix (ECM), and besides its potential role in the UPP, it has been identified as a contributor to the pathological processes in several neurological disorders. For example, Martins-de-Souza et al. (2009) showed that the expression levels of Hapln2 in the anterior temporal lobe are lower in patients with schizophrenia than those of the control subjects, and Minjarez et al. (2013) suggested that Hapln2 was probably in the neurofibrillary tangles (NFTs) from AD brain. Our studies recently revealed that Hapln2 expression levels were dramatically increased in the SN of PD patients and in the 6-hydroxydopamine-induced rat PD model (Liu et al., 2015; Wang et al., 2016). This review article summarizes recent progress focusing on the roles of Hapln2 in the central nervous system (CNS) under physiological and pathological conditions, highlighting the therapeutic potential of Hapln2 in neurological diseases.

## Hapln2 in CNS Physiology

### Hapln2 Protein Structure

The structure and roles of Hapln2 in the CNS have been studied since around the turn of this century, when an analysis of the gene now known as *HAPLN2* revealed seven exons encoding a polypeptide with an estimated molecular mass of 38 kDa (Hirakawa et al., 2000). The Hapln2 protein comprises three modules, namely, a proteoglycan tandem repeat 1 (PTR1) domain, a PTR2 domain, and an immunoglobulin-like fold (Hirakawa et al., 2000; Spicer et al., 2003). Western blotting results from Hapln2-EGFP transfected cell lysates have shown that an anti-Hapln2 antibody recognizes a band of around 55 kDa, which is in accordance with the predicted molecular mass (Wang et al., 2016). However, a band for Hapln2 of ~48 kDa is detected from SN tissue lysates of adult rats, revealing an inconsistency between the lysates from transfected cells and those from adult brain tissues (Wang et al., 2016). Given that Hapln2 was a kind of link protein, we attributed this discrepancy to hyaluronic acid modifications.

### Hapln2 Expression

The hyaluronan and proteoglycan link family of proteins consists of four members: Hapln1, Hapln2, Hapln3, and Hapln4 (Spicer et al., 2003). An amino acid sequence alignment demonstrated that these four proteins have similarities of ~52%–62% (Spicer et al., 2003), with a high level of conservation among vertebrate species. Northern analyses and EST database searches revealed that Hapln2 and Hapln4 are specifically expressed in the brain, with the levels of Hapln2 significantly higher than that of Hapln4 (Spicer et al., 2003). These data indicate that the four-link proteins may have different roles.

Northern blot analyses by Hirakawa et al. (2000) showed that *HAPLN2* mRNA is expressed at high levels in the human hippocampus, medulla oblongata, putamen, SN, thalamus, and spinal cord but at lower levels in the cerebellum, cerebral cortex, frontal lobe, and nucleus accumbens. By *in situ* hybridization, we detected high expression levels of *Hapln2* mRNA in the SN, olfactory bulb, red nucleus, cerebellum, brain stem, and hippocampus but relatively weak signals in the cerebral cortex of adult rat brain (Wang et al., 2016). The differential expression of Hapln2 among the various brain regions suggests that the protein has different roles in areas of high expression (SN and hippocampus) than in areas of low expression (cerebral cortex). The discrepancy between these studies regarding the expression in the cerebellum may reflect species specificity and/or the different experimental approaches used.

### Hapln2 in ECM Formation and Neuronal Conductivity

**Figure 1:**
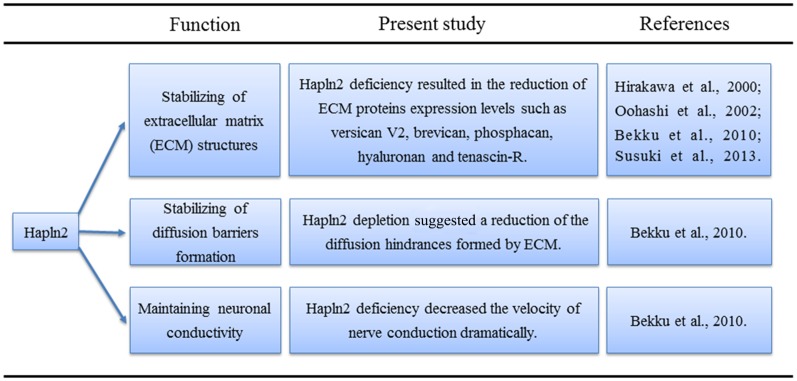
Physiological functions of hyaluronan and proteoglycan link protein 2 (Hapln2) in the central nervous system (CNS).

Lecticans such as brevican and aggrecan mainly bind to the hyaluronans, which are the major components of the ECM in the brain (Yamaguchi, 2000; Theocharis et al., 2010). The proposed function of the link proteins is to stabilize these binding interactions (Oohashi et al., 2002; Bekku et al., 2003). Northern blot analyses revealed that the expression of Hapln2 largely coincides with that of brevican, a brain-specific lectican (Hirakawa et al., 2000). Previous studies have shown that aggrecan aggregates are denser in the presence of link protein (Mörgelin et al., 1988, 1992; Oohashi et al., 2002). Moreover, immunohistochemical analyses showed that the levels of ECM proteins, such as versican V2, brevican, hyaluronan, and tenascin-R, are much lower in the brain of mice with Hapln2 deficiency (Bekku et al., 2010). These findings not only reveal the proximity of Hapln2 and lecticans in the brain but also indicate the potential role of Hapln2 in stabilizing the binding between brain lecticans and hyaluronan (Hirakawa et al., 2000; Figure 1).

Northern blot analyses have shown that Hapln2 expression in the mouse brain begins at 20 days after birth and increases with age (Hirakawa et al., 2000). Immunohistochemical analyses revealed that the patterns of Hapln2 and versican V2 expression are similar in the late developmental stage of cerebellar development and in the adult mouse brain (Oohashi et al., 2002). Moreover, Hapln2 colocalizes with versican at myelinated retinal ganglion cell axons (Oohashi et al., 2002). The Ranvier nodes could be marked by the voltage-gated Na^+^ channels antibody (Oohashi et al., 2002). Interestingly, Hapln2 preferentially localizes with versican at nodes of Ranvier in adult mouse brain, as observed by labeling with antibodies against Hapln2, voltage-gated Na^+^ channels and the contactin-associated protein (Rasband et al., 1999; Oohashi et al., 2002).

As Na^+^ channel clustering coincides with myelination (Bekku et al., 2010), it was suggested that Hapln2 may also affect the clustering of the Na^+^ channels. Of note, the clustering of Na^+^ and K^+^ channels is critical for saltatory conduction (Oohashi et al., 2002; Bekku et al., 2010). Although immunohistochemical analyses showed that Hapln2 deletion did not affect the clustering of Na^+^ and K^+^ channels, flash visual evoked potentials had a longer latency and smaller amplitude in recordings from Hapln2 knockout mice compared with those from wild-type mice (Bekku et al., 2010). Besides, consistent with the previous results mentioned above, immunostaining analysis demonstrated that there were no signals of ECM proteins including brevican, neurocan and versican at the CNS nodes in adult mice with Hapln2 deletion (Susuki et al., 2013), suggesting the essential role of Hapln2 in the formation of nodal ECM. Additionally, Susuki et al. (2013) showed that single disruption of ECM or paranodal barriers or axonal cytoskeletal scaffolds (CS) had mild effects on the development of nodes, which was consistent with the previous results to some degree (Bekku et al., 2010). However, disruption of the paranodal barriers and ECM, or paranodal barriers and CS, or ECM and CS resulted in juvenile lethality and the robust decrease of clustering of Na^+^ channels (Susuki et al., 2013), which indicates the complex and complementary roles of the three elements in the formation of nodes. Early studies showed that clustering of nodal proteins such as neurofascin-186 (NF186) was responsible for action potential (AP) propagation (Salzer, 2003; Susuki et al., 2013). Further experiments suggested that there was a specific interaction between Hapln2 and NF186 using pull-down method (Susuki et al., 2013), suggesting the important role of Hapln2 in AP propagation. All of these data indicated the important roles of Hapln2 in clustering of Na^+^ channel, nodes formation and AP propagation. However, more studies should be performed to clarify the details about the relationship between Hapln2 and the paranodal barriers or the CS in these processes during the development.

As mentioned above, Hapln2 deficiency decreased the expression of ECM-associated proteins (Bekku et al., 2010). Moreover, diffusion-weighted magnetic resonance imaging and real-time iontophoretic assays showed an increase in the diffusion signals from the ECM in white matter in animals with Hapln2 depletion (Bekku et al., 2010). What was more, Hapln2 was essential for nodal ECM formation and interacted with nodal protein NF186 directly (Susuki et al., 2013). Altogether, these results suggest that Hapln2 alters neuronal conductivity by affecting the extracellular diffusion barriers at the nodes of Ranvier rather than by directly impacting saltatory conduction (Figure 1).

## Hapln2 in CNS Pathology

### Hapln2 in PD

PD is characterized by the formation of Lewy bodies and selective loss of dopamine neurons in the SN (Liu et al., 2015; Wang et al., 2016). Despite the identification of a variety of risk factors including genetic elements, impairments in mitochondrial function and inflammatory responses, the pathogenesis of PD still remains unclear (Shao et al., 2013; Cruces-Sande et al., 2017; Billingsley et al., 2018; Segura-Aguilar and Huenchuguala, 2018). However, clinical analyses and animal experiments implicate the involvement of Hapln2 in PD. In a quantitative proteomics study, we demonstrated that the protein levels of Hapln2 were increased with the highest fold among all the upregulated proteins in the SN region of PD patients compared with the control subjects (Liu et al., 2015). This increase in the SN was confirmed both at the mRNA and protein levels by quantitative PCR and Western blotting analyses in a rat model of PD 2 weeks after 6-hydroxydopamine treatment as well as 4 weeks postlesion (Wang et al., 2016).

Flow cytometry and immunostaining analyses showed that the overexpression of Hapln2 increased the death of MES23.5 and primary neuronal cells (Wang et al., 2016). Confocal imaging of these cells demonstrated that the overexpressed Hapln2 formed aggregates, which were found to colocalize with E3 ubiquitin ligases Hrd1, Gp78, and Parkin (Wang et al., 2016). It is known that E3 ubiquitin ligases involve in specifying and catalyzing the transfer of substrates in the UPP (Uchida and Kitagawa, 2016). Moreover, the treatment of MES23.5 cells with the proteasome inhibitor MG132 increased Hapln2 levels, indicating that the UPP pathway regulates Hapln2 degradation (Wang et al., 2016). Of note, previous research showed that UPP dysfunction *via* proteasome inhibition resulted in the degeneration of dopaminergic neurons *in vitro* and *in vivo* (Kikuchi et al., 2003; Sun et al., 2006). These and the more recent results suggest that the increase in Hapln2 as a result of UPP dysfunction contributes to PD pathology.

**Figure 2:**
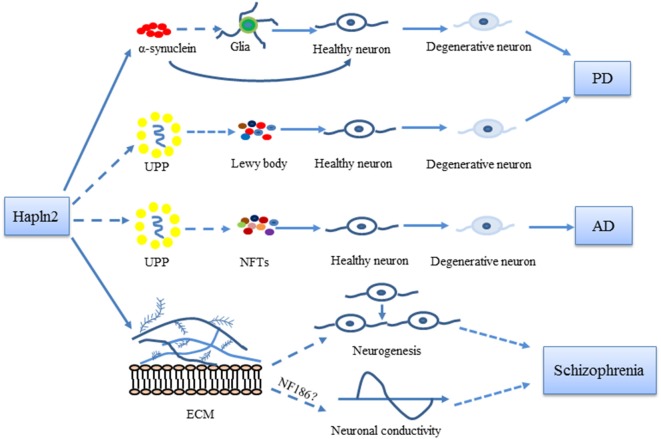
Possible roles of Hapln2 in Parkinson’s disease (PD), Alzheimer’s disease (AD), and schizophrenia. An upregulation of Hapln2 is observed in the substantia nigra (SN) of PD brain. The high expression levels of Hapln2 promote the aggregation of α-synuclein, which contributes to the degeneration of dopamine neurons and the pathophysiology of PD. Hapln2 has also been suggested in neurofibrillary tangles (NFTs). There is evidence showing the vital role of extracellular matrix (ECM) proteins during neurogenesis and neuronal conductivity, which become dysfunctional in schizophrenia. As Hapln2 helps maintain the ECM, it may also play a role in schizophrenia. Hapln2 colocalizes with several E3 ligases of the ubiquitin-proteasome pathway (UPP). The influence of Hapln2 on the ECM and UPP may contribute to PD, AD, and schizophrenia, neurological diseases in which ECM and UPP dysfunction have been observed.

Immunostaining analysis of MES23.5 cells overexpressing Hapln2 also showed an accumulation of α-synuclein, aggregates of which were found to colocalize with Hapln2 (Wang et al., 2016). Thus, Hapln2 may be another component of Lewy bodies in PD, which primarily comprise α-synuclein (Castro-Sánchez et al., 2018). The accumulation of α-synuclein not only promotes the death of dopaminergic neurons but also activates glial cells (La Vitola et al., 2018; Martinez et al., 2018). Mice overexpressing wild-type α-synuclein exhibit marked microglial activation (Su et al., 2008). Pathological α-synuclein aggregates activate astrocytes (Yu et al., 2018). Moreover, knockout of Hapln2 significantly reduces the fraction of α-synuclein that is insoluble in extracts from SN and cerebellum of 6-month-old mouse brain (Wang et al., 2016). These findings suggest that Hapln2 may contribute to neurodegeneration by regulating the activation of glial cells in the brain (Figure 2).

### Hapln2 in AD

A variety of environmental and genetic factors have also been implicated in the pathology of AD (Chin-Chan et al., 2015; Kikis, 2017; Kocahan and Doğan, 2017). However, due to the complexity of this disease, the underlying molecular mechanism remains largely unknown. Although a major hallmark of AD is the accumulation of intracellular NFTs (Manczak et al., 2018), their insolubility impedes the identification of their integral components (Perry et al., 1991; Minjarez et al., 2013). Nevertheless, Benito and colleagues used different solubilization methods to identify lots of polypeptides, including Hapln2, in total homogenates of AD brain tissue containing NFTs by tandem mass spectrometry (Minjarez et al., 2013; Sugawara et al., 2018). However, there was no direct evidence for a colocalization of Hapln2 with tau, the main component of NFTs.

Notably, both AD and PD are age-associated neurodegenerative diseases with characteristic protein aggregates (Bridi and Hirth, 2018; Daniele et al., 2018; Theofilas et al., 2018). As mentioned above, the overexpression of Hapln2 promotes the aggregate formation and is involved with neuron death and the UPP in the pathology of PD (Wang et al., 2016). As Hapln2 was found to colocalize with some E3 ligases (Wang et al., 2016), it may also contribute to AD pathology *via* disruptions of the UPP. Postmortem tissue samples from the hippocampus, superior and middle temporal gyri, parahippocampal gyri, and the inferior parietal lobes of AD patients exhibit signs of reduced proteasome activity compared with those from control subjects (Keller et al., 2000). However, further animal experiments and clinical studies are needed to determine whether Hapln2 is a major constituent of the aggregates in these diseases.

Although high levels of Hapln2 in the hippocampus were measured by *in situ* hybridization (Wang et al., 2016), the function of Hapln2 in this brain region remains unknown. The hippocampus is involved in learning and memory (Portero-Tresserra et al., 2018; Wang D. et al., 2018), the dysfunction of which is a major symptom of AD (Axelrud et al., 2018). Behavioral tests in a mouse model of AD revealed a significant impairment in contextual conditioning and performance in pattern separation tests after neurogenesis in the hippocampus was inhibited (Hollands et al., 2017). Moreover, the performance of AD transgenic mice in spatial learning and memory tasks improved when hippocampal neurogenesis was enhanced by deep brain magnetic stimulation (Zhen et al., 2017). Of note, the fate determination of neural stem cells is guided by ECM components, such as the heparan sulfate proteoglycans glypican and perlecan (Yu et al., 2017). Given the vital role of Hapln2 in maintaining the ECM scaffold (Bekku et al., 2010), whether Hapln2 is involved in AD pathology by regulating hippocampal neurogenesis needs further investigation.

### Hapln2 in Schizophrenia

Although the pathophysiology of schizophrenia has not been fully defined, genetic factors, epigenetic elements, and abnormal neurotransmission in the hippocampus are important contributors (Kim et al., 2018; Sugawara et al., 2018; Wang H. Y. et al., 2018). Previous studies of postmortem brain tissues from patients with schizophrenia revealed the presence of insoluble aggregates containing DISC1 (Atkin and Kittler, 2012; Korth, 2012). The overexpression of DISC in neuroblastoma cells leads to the formation of aggresomal deposits (Korth, 2012), which can invade cells and recruit other proteins that may contribute to the impairment of neuronal cells (Atkin and Kittler, 2012; Korth, 2012). Hapln2 represents another potential contributor, as a shotgun proteomic analysis of postmortem anterior temporal lobe tissues showed that Hapln2 protein levels are lower in schizophrenia patients than in control subjects (Martins-de-Souza et al., 2009). Additionally, a convergent pathway analysis indicated that there is dysregulation of the UPP in schizophrenia (Bousman et al., 2010). As mentioned above, the UPP is vital for protein catabolism *via* the degradation of particular substrate proteins (Tsukamoto and Yokosawa, 2006). The colocalization of Hapln2 with E3 ligases revealed in our previous work suggests a potential function of Hapln2 in the UPP (Wang et al., 2016), which suggesting that Hapln2 may also contribute to the pathophysiology of schizophrenia through dysfunction of UPP.

Moreover, it has been reported that ECM plays important roles in regulating neurogenesis, axonal outgrowth, synaptogenesis and cell migration (Bandtlow and Zimmermann, 2000; Faissner et al., 2010; Gundelfinger et al., 2010). The analysis of the schizophrenia risk genes showed that most of the genes were involved in the regulation of neuronal migration and cell adhesion (O’Dushlaine et al., 2011; Lips et al., 2012; Aberg et al., 2013). Additionally, the disruption of conduction velocity has been suggested in patients of schizophrenia (Thaker, 2008; Takahashi et al., 2011). The study of schizophrenia animal models indicated the reduction of conduction velocity, indicating the important role of nerve conduction in schizophrenia (Roy et al., 2007; Tanaka et al., 2009; Takahashi et al., 2011). As one of the important components of ECM, Hapln2 may be involved in the pathogenesis of schizophrenia through regulating the neuronal migration and velocity of nerve conduction. More studies should focus on the molecular mechanism of Hapln2 in the pathogenesis of schizophrenia using animal models and clinical analysis.

## Conclusions

As a brain specific-hyaluronan and proteoglycan link protein, Hapln2 plays vital physiological roles in the formation and maintenance of the ECM scaffold. Studies in knockout mice have also revealed that Hapln2 influences neuronal conductivity. Several studies have revealed the involvement of Hapln2 in the pathogenesis of neurological diseases including PD, AD and schizophrenia, which provided new insights into the underlying molecular mechanisms of brain disorders (Figure 2).

Current research suggested that Hapln2 was involved in the formation of α-synuclein aggregates in PD pathology and study in AD suggested that Hapln2 might be the component of NFTs aggregates (Minjarez et al., 2013; Wang et al., 2016). It is noteworthy that, besides PD, AD and Schizophrenia, dysfunction of UPP system has also been implicated in some other neurological diseases including Huntington’s disease (HD) and amyotrophic lateral sclerosis (ALS; Zhao et al., 2016; Desai et al., 2018; Harding and Tong, 2018; Thibaudeau et al., 2018). Thus, it is likely that Hapln2 may also be involved in the pathological processes of these diseases through the UPP system. However, further studies of animal and clinical experiments are needed to clarify these mechanisms and the precise role that Hapln2 plays in these neurological disorders in the future. Nevertheless, the restriction of Hapln2 expression to the brain suggests that it is an important contributor.

## Author Contributions

QW, CW, BJ, and CY wrote the manuscript. JC and JZ edited the manuscript. All the authors approved the final manuscript.

## Conflict of Interest Statement

The authors declare that the research was conducted in the absence of any commercial or financial relationships that could be construed as a potential conflict of interest.
